# Coal-straw co-digestion-induced biogenic methane production: perspectives on microbial communities and associated metabolic pathways

**DOI:** 10.1038/s41598-024-75655-z

**Published:** 2024-11-04

**Authors:** Sohail Khan, Ze Deng, Bobo Wang, Zhisheng Yu

**Affiliations:** 1 College of Resources and Environment, University of Chinese Academy of Sciences, 19 A Yuquan Road, Beijing, 100049 P. R. China; 2 PetroChina Research Institute of Petroleum Exploration and Development, Beijing, 100083 P. R. China; 3 RCEES-IMCAS-UCAS Joint-Laboratory of Microbial Technology for Environmental Science, Beijing, 100085 P. R. China; 4 College of Resources and Environment, University of Chinese Academy of Science, 19 A Yuquan Road, Beijing, 100049 P. R. China

**Keywords:** Coal, Biomethane, Microbial communities, Anaerobic co-digestion, Metabolic pathways, Metabolites, Biotechnology, Microbiology, Environmental sciences

## Abstract

This study assessed the impacts of wheat straw as a cosubstrate on coal biocoverion into methane and the associated mechanism within methane metabolic pathways. Co-digestion of coal with varying wheat straw concentrations resulted in a remarkable (1246.05%) increase in methane yield compared to that of the control (CK). Moreover, microbial analysis revealed a uniform distribution of *Methanosarcinaceae* (51.14%) and *Methanobacteriaceae* (39.90%) in the co-digestion of coal and wheat straw (CWS1) at a ratio of 3:1 (w/w) compared to other treatments such as coal and wheat straw (CWS2) at a ratio of 3:0.5. In addition, *Hungatieclostridiaceae* and *Rhodobacteriaceae* were abundant in both co-digesters, whereas the bacterial communities in the CK group were significantly different and more abundant than those in the *Peptostreptococcaceae* and *Enterobacteriaceae groups.* The key enzymes related to methanogenic metabolic pathways, including EC: 1.2.99.5 and EC: 2.1.1.86 (facilitating the conversion of CO_2_ into methane), and EC:1.12.98.1 exhibited significant abundance within CWS1. Aromatic compounds such as 4-(2-chloroanilino)-4-oxobutanoic acid and phthalic acid were substantially more abundant in CWS1 and CWS2 than in CK, indicating the increased bioavailability of coal to microbial activities. This novel approach demonstrates that wheat straw co-digestion with coal during anaerobic digestion modulates microbial communities and their metabolic pathways to enhance methane production from complex substrates such as coal.

**Supplementary Information:**

The online version contains supplementary material available at 10.1038/s41598-024-75655-z.

## Introduction

Biogenic methane is an essential component of coal seam resources generated by anaerobic microorganisms using coal as a carbon source^1^. Microbial biodegradation of coal remains challenging due to its complex organic fractions of aromatic, aliphatic, and lignin-derived macromolecules^2^. However, the low bioavailability of organic matter in coal, such as benzene, alicyclic, and heterocyclic structures, strictly limits methane generation rates and yields^3^. Several pretreatments, such as physical, chemical, and biological methods, have been applied along with the addition of trace metals to enhance methane production from coal^4, 5^. Several studies have shown a 37% increase in methane production with a suitable dosage of trace metals^6–9^. The commercial viability of these techniques is limited because they facilitate groundwater pollution and economic restrictions^10^. Moreover, the low H/C ratio of approximately 1:24 in coal results in unfavorable conditions for the production of biogenic methane by microorganisms^11^.

Recently, co-digestion of two or more substrates from complex organic compounds has been considered a sustainable and economically viable approach for biogas production^12^. Straw biomass, which is mainly composed of lignocellulosic components and is rich in H/C, is often used for biogas production^13^. Straw co-digestion with coal can increase the H/C ratio to increase biomethane generation^12^. Limited studies are available on the co-digestion of coal and straw biomass. Yoon et al. explored methane enhancement by co-digesting rice straw with lignite and reported an increase in methane production with a rice-to-lignite mass ratio greater than 33.3%^14^. Moreover, the co-degradation of maize straw with lignite and two bituminous coals has enhanced methane production by 448.98% compared to coal degradation alone^12^. The addition of corn straw was also reported to exhibit beneficial effects on archaeal genera and diminish unwanted bacteria compared with coal digestion alone^15^.

Moreover, methane metabolism is a prevalent process during the anaerobic degradation of organic substrates and biogas production. Therefore, it is necessary to investigate the metabolic characteristics of microbial communities during methane production^16^. Methane metabolic pathways during the anaerobic digestion of various organic substrates have been previously studied. For instance, methane generation from acetic acid was found to be the most prominent pathway compared to methane generation from CO_2_ and H_2_ during the anaerobic digestion of municipal solid wastes (Ma et al.). In addition, syntrophic relationships have also been detected among acetate-oxidizing bacteria (AOB) methanogens. The oxidation of acetate to H_2_ and CO_2_ occurs by these bacteria, which are then utilized by hydrogenotrophic methanogens for methane generation (Ghosh et al.). Generally, the efficiency of anaerobic digestion depends on the syntrophic association of acidogenic, and methanogenic bacteria based on their metabolic pathways (Wang et al.). The most important methanogenic pathways include acetoclastic (acetate), hydrogenotrophic (H_2_, CO_2_), and methylotrophic (methyl substrate and H_2_) pathways (Cai et al.). However, the impacts of wheat straw as co-substrate with coal on methane production, the generation of metabolic intermediates, the microbial communities, and the related methanogenic pathways remains unknown in the co-digestion process. Therefore, investigating these aspects in coal and wheat straw co-digestion would provide valuable insights for enhancing methane production. Hence, it is of foremost importance to oversee the microbial composition and analyze metabolic pathways to augment methane generation by enhancing the activities of important microorganisms during the co-degradation process. The functional enzyme activities associated with anaerobic microorganisms have not been previously investigated in the anaerobic digestion of coal and wheat straw co-digestion.

Hence, this study was conducted to evaluate the impacts of using wheat straw as a co-substrate on microbial diversity, microbial metabolic characteristics and associated functional metabolic pathways during methane production from lignite coal. In addition, LC‒ESI‒MS/MS-based analysis was conducted on the succession of metabolic products at the decline phase of the experiment. This investigation presents the groundwork for an inclusive understanding of promoting biogenic methane generation and metabolism by supplying wheat straw during coal digestion, thereby increasing the potential application of coal-straw co-conversion into methane.

## Materials and methods

### Coal sample and source of inoculum

Coal samples were collected aseptically in sterilized bottles from the Huaibei Coalfield, Anhui Province, and stored at 4 °C under anaerobic conditions. The coal samples were then crushed via a morter pestle and sieved through a 200-mesh seiver to collect coal particles with a diameter of 250 μm. The ultimate and proximate analyses of the coal were performed by the test center of the China Coal Research Institute in Beijing (Tables 1 and 2). In addition, ground straw biomass residues remaining in the mesh sieve were used as cosubstrates after pretreatment with 1.6% (w/w) NaOH and H_2_SO_4_ and subsequently neutralized with distilled water and drying. However, alkali and acid pretreatment can effectively breakdown lignin and hemicellulose to expose cellulose for further degradation by hydrolytic bacteria^17^. Lignin works to support the structures of plants and to prevent the permeation of microorganisms and subsequent detoxification^18^.

**Table 1:** Proximate analysis of coal samples used for biomethane production.

Proximate analysis	M_ad_ %	A_ad_ %	AD %	V_ad_ %	Vd %	V_daf_ %	F_cad_ %
Coal	12.66	41.47	47.47	22.06	25.26	48.08	23.82

**Table 2:** Ultimate analysis of coal samples used for biomethane production.

Ultimate analysis	C_ad_ (%)	H_ad_ (%)	*N*_ad_ (%)	S_ad_ (%)
Coal	28.24%	2.06%	0.46%	0.34%

Note: *M*, moisture; *A*, ash yield; *V, *volatile matter; ad, air-dry basis; daf, dry ash free; Fc, fixed carbon; *C,* carbon; *H, *hydrogen; *N,* nitrogen; *S, *sulfur.

Microbial inoculum was obtained from coal formation water after approximately 5 L was filtered through 0.22 μm pore size Whatman filter paper (Whatman Japan KK, Japan). These filter papers adhering to microbes were used for coculture with 10 mL of sludge in the prepared medium for enrichment under anaerobic conditions.

### Media preparation and enrichment

Anaerobic media were prepared for inoculum enrichment as described previously by Wang et al. (2018). The ingredients used in the medium were (g/L) MgSO_4_.7H_2_O (3.45), KCl (0.335), NH_4_Cl (0.25), NaCl (11.00), MgCl_2_.2H_2_O (2.75), K_2_HPO_4_ (0.14), and CaCl_2_.2H_2_O (0.14). Moreover, 40 mL of 1.25% Na2S, 1.25% cysteine, 1 mL of 0.2% Fe(NH_4_)2(SO_4_)2, 10 nM HEPES buffer (pH 7.5), 10 mL/L vitamin and 10 mL/L trace metal solution were added to the medium. The vitamin and trace metal solutions contained the following in mg/L: pyroxidine HCl (10), folic acid (2), biotin (2), riboflavin (5), thiamine HCl (5), lipobenzoic acid (5), vitamin B12 (0.1), nicotinic acid (5), lipoic acid (5), ZnCl_2_ (7), Cucl_2_ (0.2), H_3_BO_3_ (1), AlK(SO_4_)_2_ (1), NaMoO_4_ (0.6), FeCl_2_.4H_2_O (0.15), NiCl_2_.6H_2_O (2.4), MnCl_2_.4H_2_O (10), CoCl_2_.6H_2_O (19).

### Inoculum preparation, bioreactor setup and operation

After sterilizing the media at 121 °C for 15 min, the filter papers adhering to microbes from the formation water were cut into pieces and introduced into the media under anaerobic conditions. The bottles containing the medium were flushed with 99.9% N_2_ for two minutes to ensure an anaerobic environment in the reactors. Thereafter, the bioreactors were incubated at 35 ± 2 °C for 6 days to enrich the microorganisms, followed by daily methane detection. The enriched medium was then transferred to the working reactors labeled CK, which contained only coal at a total concentration of 10 g. CWS1 and CWS2 contained coal and straw biomass at ratios of 3:1 (w/w) and 3:0.5 (w/w), respectively, in 250 mL serum bottles with a working volume of 200 mL. The total duration of the experiment was 21 weeks, and the experiment was divided into three phases. After the first and second phases, the media of the reactors were replaced with sterilized media containing the following ingredients in g/L: NH_4_Cl (0.6), MgCl_2_.6H_2_O (0.1), K_2_HPO_4_.3H_2_O (0.32), KH_2_PO_4_ (0.16), yeast extract (1), Na_2_S (0.7), and tryptone (0.1). All the cultivations were performed in triplicate and manually agitated three times a day.

### Headspace gas measurement and analysis

The accumulated biogas in the head space was measured through a 100 mL syringe every week, and the methane concentration was determined using 1 mL of gas withdrawn from the headspace and injected into a gas chromatograph system (Agilent 7890 A; Agilent Technologies, USA) equipped with a flame ionization detector. The peak areas obtained for the GC samples were compared with the standard peak to accurately quantify the concentrations of methane.

### DNA extraction and sequencing

For the extraction of DNA and sequencing, mixed liquid samples (2 mL) were collected from each reactor at the end of the experiment. A Fast DNA spin kit (Bio101 Systems, USA) was used to extract genomic DNA from the samples according to the manufacturer’s instructions^19^. The archaea-specific primer set AR-519 F/915R and bacteria-specific primer set BAC-515 F/907R were employed to determine the microbial communities by amplifying 16 S rRNA in the V3 and V4 regions. PCR products were identified via agarose gel electrophoresis after quality inspection and library construction. A magnetic bead nucleic acid purification kit was used to recover the PCR products. DNA was quantified using a Qubit 2.0 DNA detection kit, and HiSeq2500 PE250 was subsequently used for sequencing^20^. To assess the metabolic potential of microbial communities CK, CWS1, and CWS2, PICRUSt software (community phylogenetic investigation conducted by reconstructing the unabsorbed state) and KRGG (Kyoto Encyclopedia of Genes and Genomes) database was used to predict the functional gene content of the microbial communities shown is Greengenes database of 16 S rRNA gene sequences^21^.

### Liquid chromatography‒mass spectrometry (LC‒MS)

Metabolites produced during methane formation in coal combined with straw biomass were detected by LC‒MS as described in previous studies^19^. Specifically, an Agilent 1290 Infinity II UHPLC system combined with an Agilent 6545UHD and Accurate-Mass Q-TOF/MS was used for LC‒MS detection. A Vydamas^®^ C19-P 100 A column (5 μm, 250 mm*2.1 mm) was used as the chromatographic column. Mobile phase: A: aqueous solution with 0.1% formic acid. B: acetonitrile solution. Flow rate: 0.2 mL/min. injection volume: 10 µl. The column temperature was 296 K. The gradient elution conditions were as follows: 0–0.5 min, 0% B; 0.5–4 min, 0–15% B; 4–5 min, 15% B; 5–20 min, 15–95% B; 20–21 min, 95 − 0% B; and 21–23 min, 0% B. The post exposure time was 6 min for system equilibration. Mass spectrometry was performed in both positive and negative ion modes. The ion source used was electrospray ionization. The optimized parameters were set as follows. The drying gas flow rate was 10 L/min. Capillary voltage: 4 kV. Nebulizer pressure: 20 psig. Skimmer voltage: 45 V. Fragmentor voltage: 120 V. Ion source temperature: 400 °C. Mass range: m/z 50-1000. Reference ions were used to ensure mass accuracy during the MS acquisition process. The reference ions in positive ion mode were 121.0509 and 922.0098. The reference ions in negative ion mode were 112.9856 and 1033.9981.

#### Metabolomic analysis

Agilent Masshunter Qualitative Analysis B.08.00 software (Agilent Technologies) was used to convert the raw data to the common (mz. Data) format. In the R software platform, the XCMS program was used to perform automatic retention time correction integration pretreatment and peak identification. Visualization matrices containing the sample name, peak area, and m/z RT pair were acquired. The peak areas in the chromatograms were used for relative content analysis in the present study. The qualitative metabolites were searched in the Metlin online database for accurate molecular weight comparisons^22^. Adduct manner: [M + H] + was selected in positive mode, [M-H]^_^ was selected in negative mode. Mass error value: 30 ppm. Metaboanalyst 4.0 was used for comprehensive and integrative metabolomics data analysis^23^.

## Results and discussion

### Biogenic methane generation from coal mixed with straw biomass

**Figure 1:**
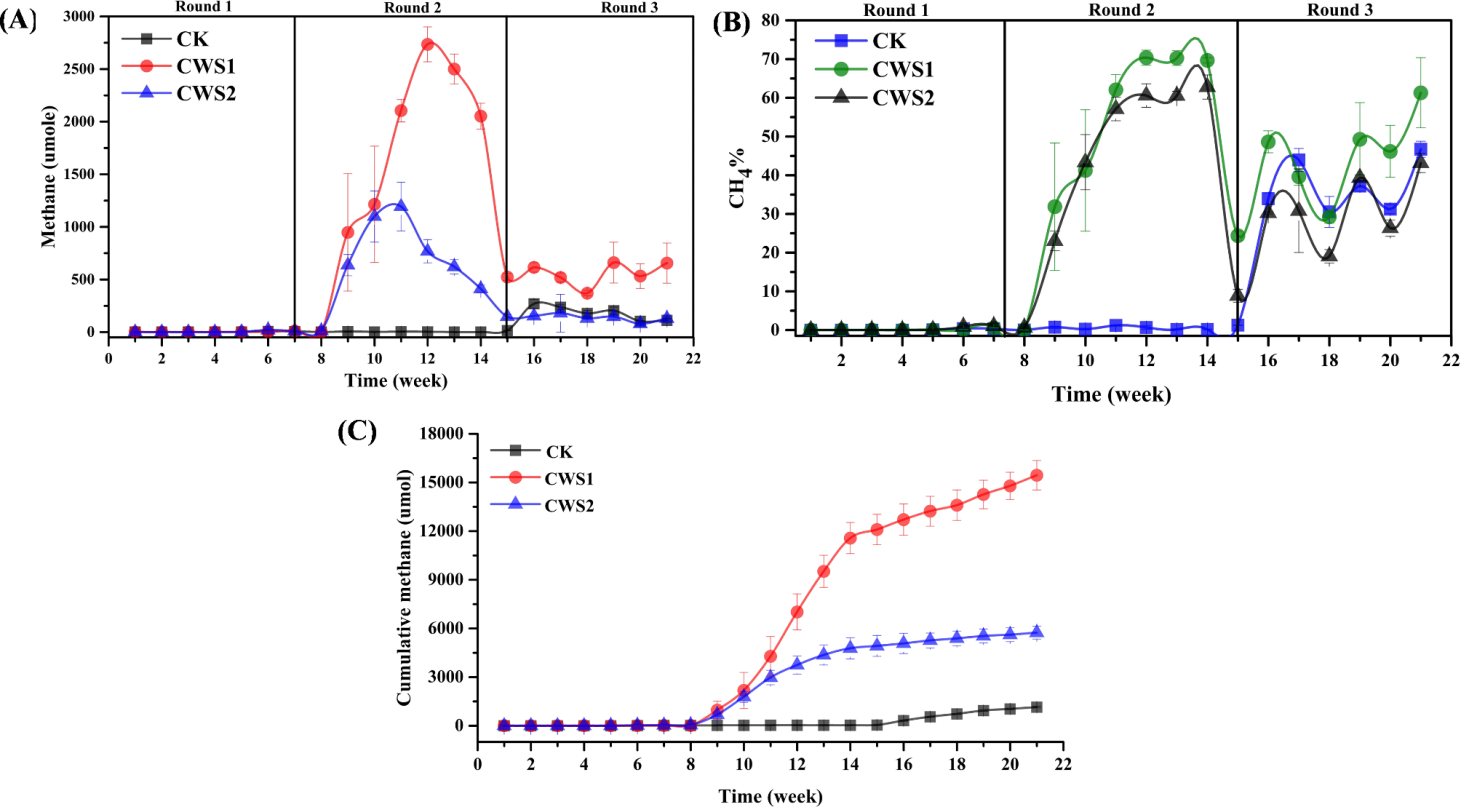
(**A**) Daily methane production, (**B**) methane contents in biogas produced from mixed coal and straw biomass anaerobic co-digestion during 21 weeks of cultivation. and (**C**) cumulative methane production, Error bars represent the standard deviation for replicate reactors.

The anaerobic co-degradation of coal with organic carbon-rich materials under the right circumstances can generate significant amounts of biogenic methane^24^. As shown in Fig. 1, the co-digestion of coal with different concentrations of straw biomass promoted biomethane production. The coal combined with straw biomass in CWS1 produced 2734.45 µmol of methane, followed by CWS2 (1193.32 µmol) and CK (268.55 µmol) (Fig. 1A). Additionally, more than 70% of the methane content was detected in the biogas from CWS1, followed by CWS2 (62.77%) and CK (46.68%) (Fig. 1B). Unexpectedly, wheat straw mono-digestion did not result in significant methane production (results not included), as straw biomass has been widely reported to generate more methane during the anaerobic digestion process. One possible reason could be the size of the straw particles, as large particles were used in this study, which could limit their surface area for microbial degradation^18^. On the other hand, the high methane production during co-digestion could be due to the balanced nutrients provided by each substrate.

Previous studies on the co-digestion of lignite with rice straw and corn straw and on bituminous coal with corn straw have shown that different types of straw have different effects on coal co-degradation and methane production^12, 14, 25^. Moreover, higher methane production was observed in the present study, which could be due to the enhanced co-metabolic process of wheat straw and lignite coal and the structural composition of organics in these two substrates. Furthermore, the lag phase of the co-digestion process for methane production was 49 days, which might be the cause of volatile fatty acid (VFA) accumulation^26^. As VFAs accumulation decreases the pH of the fermentation liquid, which inhibits methanogenic activities^12^. It could also be possible that the dynamics of the microbial community do not represent any neutral transition that leads to process delay^27^.

An increase in methane production was observed after the 56th day of the process, when the reactors were refreshed with medium, and the methane production linearly increased until the 84th day. Interestingly, an increase in methane yield from single coal digestion (CK) occurred in the third phase of the experiment with increasing methane purity. This increase might be possible after the long interaction of coal with bacterial communities to extract metabolites from it, which are then utilized by methanogens to generate methane. This phenomenon also shows that reactors refreshed with medium replenish essential nutrients that may have depleted over time. Which supports the growth and metabolic activities of microorganisms, predominantly methanogens, thereby promoting methane production.

Moreover, the high concentration of straw biomass with coal resulted in high cumulative biomethane generation in CWS1 (15444.02 µmol), followed by CWS2 (5738.26 µmol) and CK (1147.38 µmol) (Fig. 1C). The higher methane yield in CWS1 could be supported by the fact that straw biomass containing more cellulose and hemicellulose is more likely to play an important role in improving methane generation during co-degradation^14^. In addition, the increased amount of straw biomass with coal can maintain the populations of methanogens by providing easily degradable organics with essential nutrients that could maintain the methanogenic activities^14^.

### Microbial commuunity analysis

**Figure 2:**
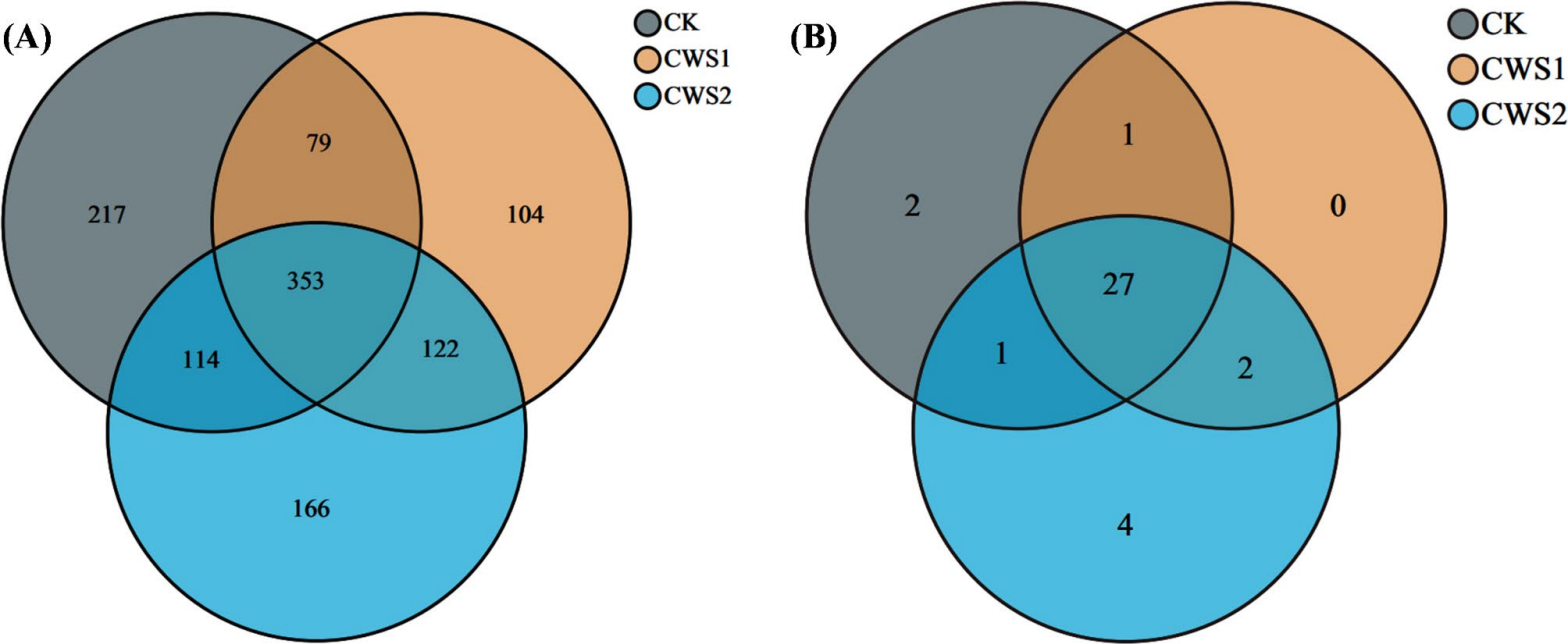
Venn diagram representing the number of OTUs between coal co-digested with straw biomass (CWS1 and CWS2) and coal mono-digested (**a**) the bacterial community and (**b**) the archaeal community.

**Figure 3:**
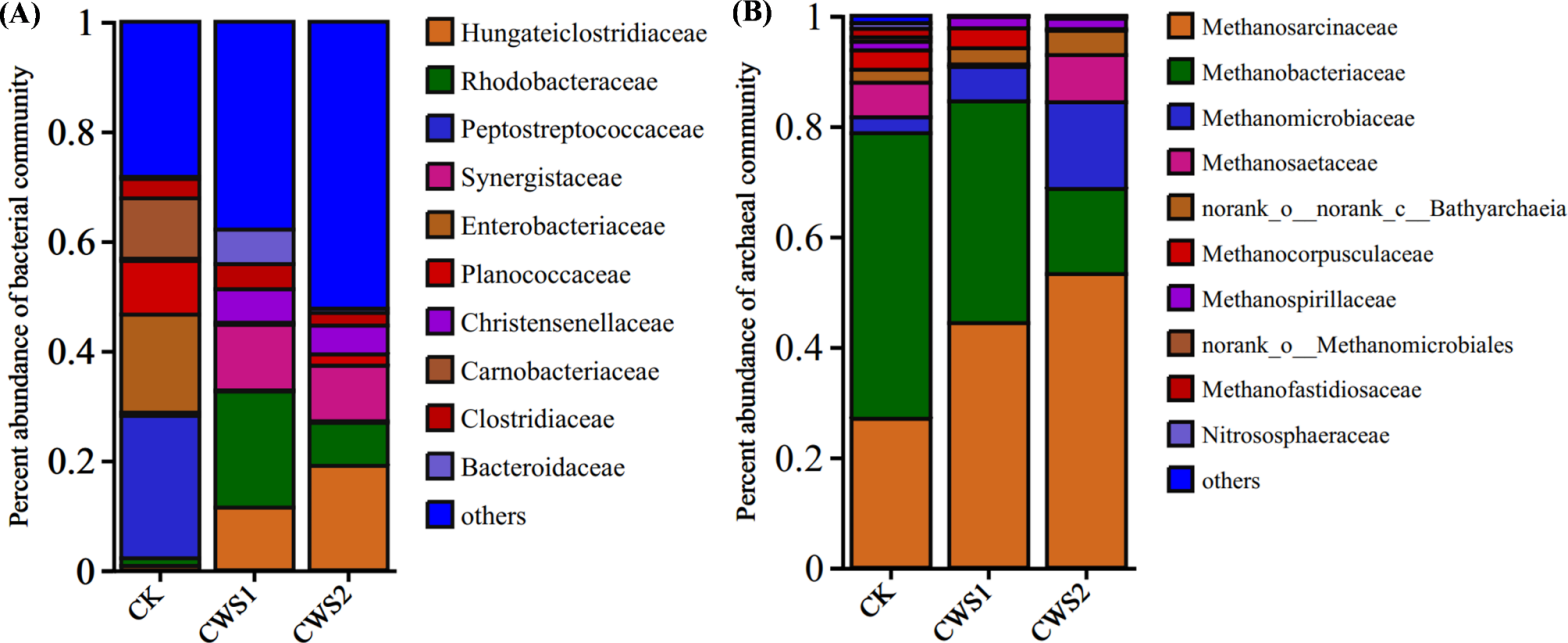
Differences in the abundances of microbial communities (**A**) Bacterial community and (**B**) Archaeal community at the family level between coal mono-digestion and coal co-digestion with straw biomass.

An Illumina HiSeq2500 PE250 system was used for 16 S rRNA gene analysis. Differences in both the bacterial and archaeal community structures among CK, CWS1, and CWS2 were observed through Venn diagram analysis (Figs. 2 and 3). For bacteria, a total of 353 OTUs were shared among all the reactors. The most unique OTUs were observed in CK (217 OTUs), followed by CWS1 (166 OTUs) and CWS2 (104 OTUs) (Fig. 2a). For archaea, a total of 27 OTUs were shared among all the groups, while 4 unique OTUs were found in CWS1, and 2 OTUs were found in CK. In addition, CWS2 had 0 unique OTUs (Fig. 2b). These results show that the bacterial communities were more affected in all the reactors. These results also indicate that wheat straw as co-substrates added with coal at different concentrations could alter the microbial communities and further impact the methane generation.

Variance among the bacterial and archaeal communities was detected in the different reactors via NMDS analysis (Fig. S1). NMDS analysis revealed that the shorter the distance between two sample points was, the smaller the variability in the community and vice versa. In the present study, the differences in the bacterial (Fig. S1a) and archaeal communities (Fig. S1b) were large, indicating that the community structure was affected during the anaerobic co-digestion of straw biomass and coal.

#### The structure of bacterial community

Figure 3 represents the abundance of the dominant microbial communities in each reactor. The family-level dominant bacterial community in the coal-digesting reactor (CK) was *Peptostreptococcaceae* (25.95%), followed by *Enterobacteriaceae* (17.87%), *Carnobacteriaceae* (10.99%), *Planococcaceae* (9.77%), and *Clostrideaceae* (3.5%) (Fig. 3A). *Peptostreptococcaceae* within the order *Clostridiales* have been reported to use hydrocarbon intermediates to produce volatile fatty acids such as acetic acid and formic acid^28^. The family *Enterobacteriaceae*, belonging to the classes *Clostridia* and *Gammaproteobacteria*, has been reported to produce H_2_, CO_2_, acetate, butyrate, propionate and lactate or ethanol^29^. In addition, these bacteria play an essential role in lignite degradation due to their lignocellulolytic activity^30^. *Carnobacteriaceae* and *Planococcaceae*, on the other hand, have been reported to be abundant under iron and nitrate-reducing conditions, respectively. These bacteria can use nitrate and iron as electron acceptors and have been reported to promote hydrogenotrophic methanogen growth during phenanthrene biodegradation^31^. This phenomenon was observed in the present study, as hydrogenotrophic methanogens such as *Methanobacteriaceae* were abundant in the CK reactor.

In addition, the bacterial composition in the coal co-digested with wheat straw was significantly different from that in the CK. The bacterial families in CWS1 were dominated by *Rhodobacteriaceae* (21.15%), *Synergistaceae* (11.95%), *Hungatieclostrideaceae* (11.43%), *Bacteroidaceae* (6.28%), *Christensenellaceae* (6.11%), and *Clostrideaceae* (4.57%). The relative abundance of bacteria in the CWS2 reactor was dominated by the genera Hungatieclostrideaceae (19.48%), *Synergistaceae* (10.11%), *Rhodobacteriaceae* (7.85%), *Christensenellaceae* (5.24%), *Clostrideaceae* (2.31%), and *Planococcaceae* (2.04%) (Fig. 3A). All these bacterial taxa have been shown to play a positive role in the biodegradation of organic matter and have also been reported in coal formation water and anaerobic digesters^14, 27, 32–39^. The prevalence of these genes can be related to the dissolution of transitional compounds from both substrates according to a spectrum of genes, such as *bssA*, *bamA*, *bamB*, *bzdN*, *bcrA*, and *bcrC*^40, 41^. Specifically, the most prevalent *Rhodobacteraceae* family is a type of heterotrophic bacteria with a high degree of metabolic diversity that is capable of metabolizing a diverse range of hydrocarbons, including dimethylsulfoniopropionate^42^. For instance, *Rhodobacter* was identified in the Illinois Basin and characterized as a type of hydrogen-producing bacterium with an unknown mechanism^43^. Similarly, the relative abundance of the *Hungateiclostridiaceae* family increased, suggesting the generation of volatile fatty acids (VFAs) by degrading hydrocarbons^44^. In addition, *Synergistaceae* have been reported previously in straw-fed reactors and are known to be syntrophic acetic acid oxidating bacteria (SAOB) that play an important role in building syntrophic associations with methanogens during anaerobic digestion^45^. These results indicate that all the detected bacteria may be involved in the primary degradation of coal and straw and their conversion into such compounds that are subsequently utilized by other microbes. Overall, the high methane production in the co-digestion process might be due to the addition of straw biomass, which influences and shapes the potential microbial communities leading to high biodegradation of organic matter.

#### Archaeal community analysis

*Euryarchaeota* was the predominant phylum in the three different reactors, accounting for 99% of the whole methanogenic community. Among the methanogenic communities, the relative abundance of *Methanosarcinaceae* was significantly greater in CWS2 (51.14%) than in CWS1 (42.95%) and CK (26.99%). In addition, *Methanobacteriaceae* were more abundant in CK (51.72%) than in the other reactors, such as CWS1 (39.90%) and CWS2 (16.04%) (Fig. 3B). Moreover, the abundances of the families *Methanomicrobiaceae* and *Methanosaetacea* in CWS2 were 16.19% and 9.70%, respectively.

The substantial methane production in CWS1 might have resulted from the even distribution of these two methanogens, i.e., *Methanosarcinaceae* and *Methanbaceriaceae*, allowing the optimal utilization of nutrient substrates in the system. However, the abundance of *Methanosarcinaceae* in our findings is consistent with other reported studies showing that members of this family represent a large proportion following culture enriched with coal^46^. Moreover, it is a representative acetotrophic methanogen, and its prevalence in the co-digestion process suggested that high methane generation was strongly associated with *Methanosarcinaceae*. *Methanosarcinaeae* can metabolize a wide array of substrates, including acetate, methanol, methylamine and H_2_^27^. On the other hand, *Methanobacteriaceae* can generate methane by using formate, H_2_/CO_2_ gas mixtures and simple alcohols^47^. These methanogens constitute the main methanogenic flora and play an essential role in the anaerobic biodegradation of organic matter as terminal metabolic groups^48^.

### Methanogen metabolic pathway determination

**Figure 4:**
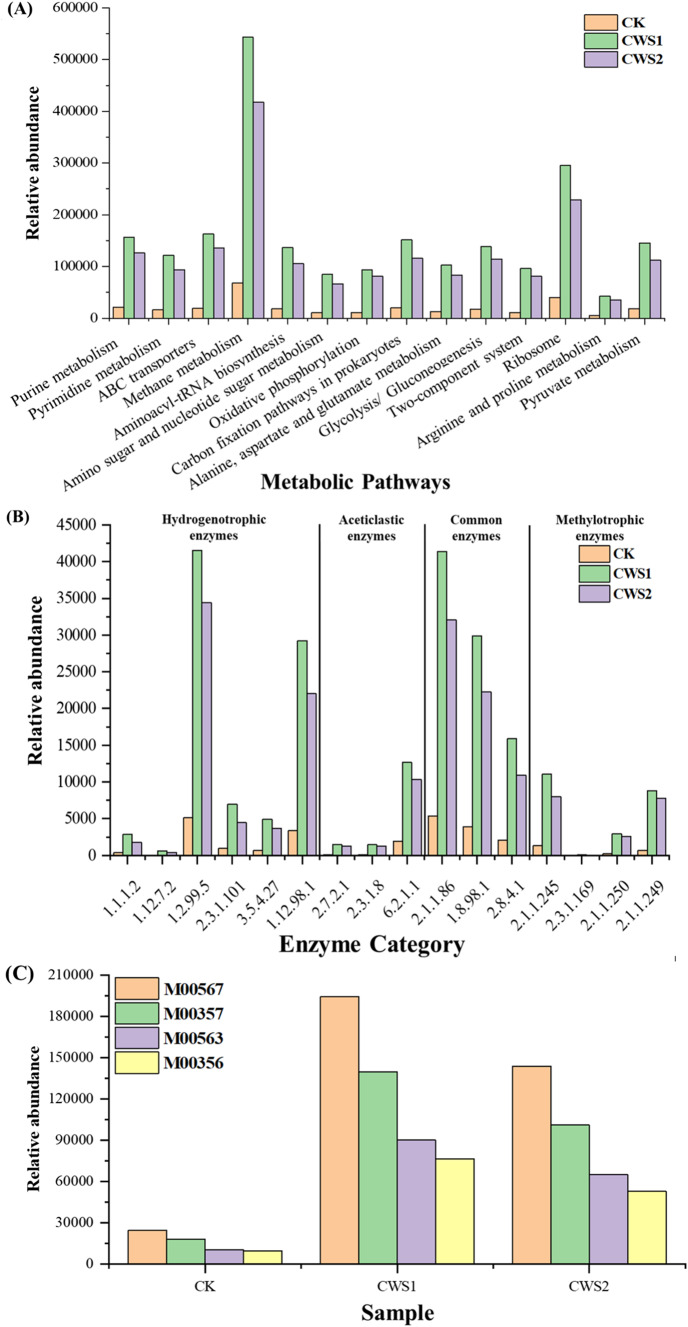
(**A**) Abundance of enriched metabolic pathways, (**B**) Metabolic enzymes participating in methanogenesis, and (**C**) enriched modules involved in methanogenesis.

To understand the methanogenic metabolic process, the KEGG database was used to explore the process via PICRUS software. The corresponding metabolic pathways involved in methanogenesis are shown in Fig. 4a, where methane metabolism (ko00680) was the dominant pathway in all the reactors, followed by ribosome metabolism (ko03010), ABC transporters (ko02010), and purin metabolism (ko00230). Most importantly, the abundances of all these pathways were significantly greater in CWS1 than in CWS2 but were lowest in CK. It has been reported that ABC transporters play essential roles in nutrient uptake and can eradicate toxins^49^. Moreover, bacteria can modify themselves according to conditions through their nutrient uptake and transport systems^50^.

**Figure 5:**
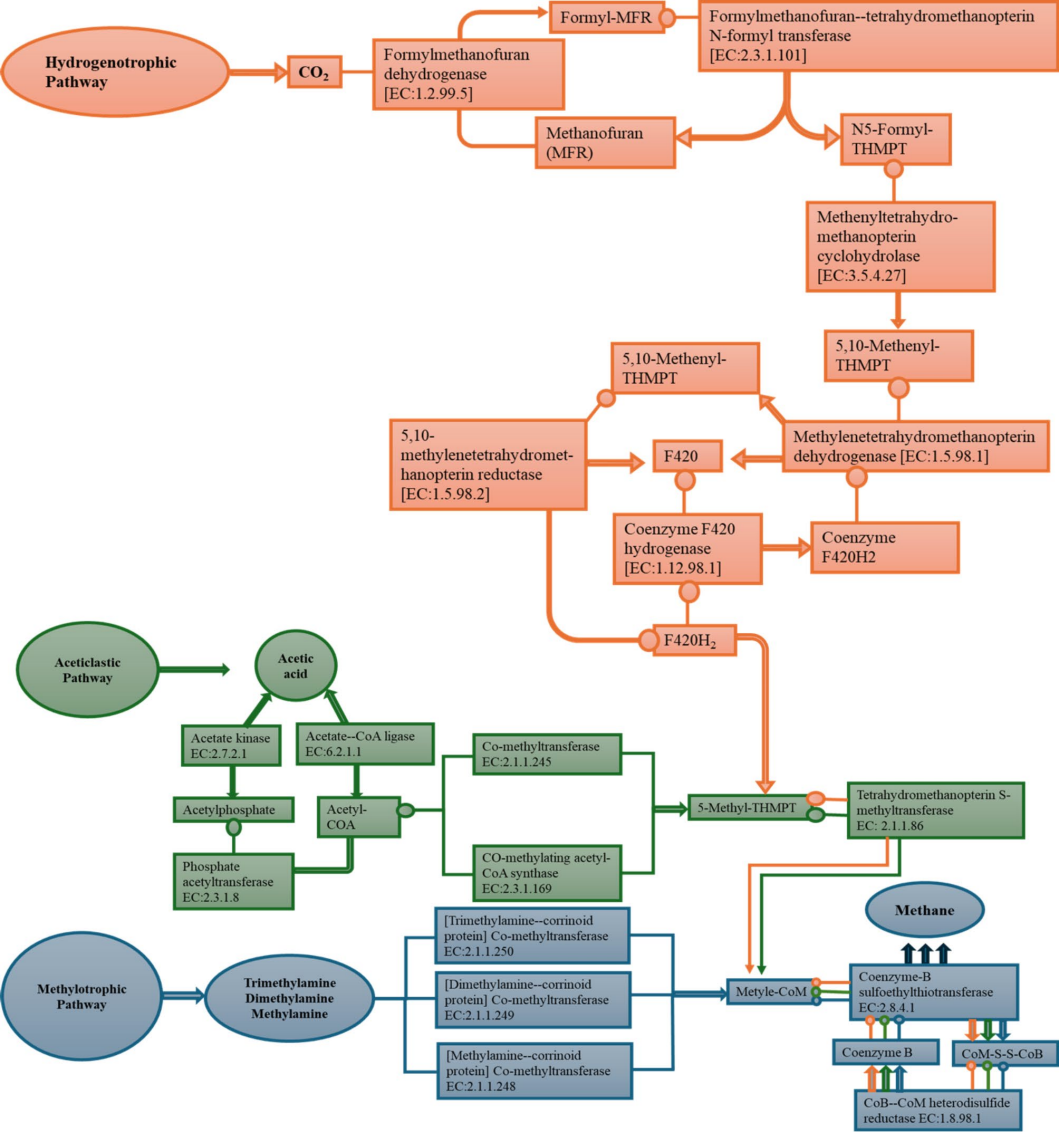
Methane metabolic pathways based on KEGG analysis. The Pathway map was generated using KEGG database identifiers. The data for KEGG pathway were retrieved from the Kyoto Encyclopedia of Genes and Genomes (KEGG), Kanehisa Laboratories. Available at: www.kegg.jp/kegg/kegg1.html^51^.

Furthermore, as shown in Fig. 4b, essential enzymes responsible for methanogenesis was consistent with other reported studies^32, 33, 49, 50^. The most prominent bacterial and archaeal essential enzymes in the three different treatments are depicted in Fig. S2. Methanogens can generate methane via three main pathways according to substrate availability: the hydrogenotrophic, aceticlastic and methylotrophic pathways^34^. The hydrogenotrophic pathway was enriched with formylmethanofuran dehydrogenase (EC: 1.2.99.5) and coenzyme F420 hydrogenase (EC:1.12.98.1). However, supplementation with higher concentrations of straw biomass (CWS1) significantly increased the abundance of these enzymes (Fig. 4b). Formylmethanofuran dehydrogenase is an oxidoreductase that acts on the aldehyde or oxygen groups of donors, with ferritin serving as a receptor to convert CO_2_ into CH_4_^50^. In addition, the coenzyme F420 hydrogenase is also considered a type of oxidoreductase that can bind to other known receptors with hydrogen as a donor. It has been anticipated that the high abundance of *Methanobacteriaceae* in the methanogenic community may involve a hydrogenotrophic pathway to facilitate this conversion. Initially, acetic acid can be converted to acetyl-CoA in the aceticlastic pathway^33^. In the aceticlastic pathway, enzymes such as acetate-CoA ligase (EC: 6.2.1.1) was significantly greater in CWS1 than in CWS2 and CK. Acetate-CoA ligase can be used by *Methanosaetaceae* to facilitate the conversion of acetate to acetyl-CoA^35, 36, 50^. However, in this study, the abundance of *Methanosaetaceae* was lower in CWS1 than in CWS2 and CK (Fig. 3b). Therefore, the high abundance of acetate-CoA ligase might be related to methanogens other than *Methanosaetaceae* identified in this study. Common enzymes from the three methanogenic pathways known as tetrahydromethanopterin S-methyltransferase (EC: 2.1.1.86), CoB-CoM heterodisulfide reductase (EC: 1.8.98.1), and Coenzyme-B sulfoethylthiotransferase (EC: 2.8.4.1) have been reported to be involved in many of the last steps of methane generation^37^. Tetrahydromethanopterin S-methyltransferase is a methyltransferase involved in the transfer of carbon-containing groups and contributes to the transformation of CO_2_ into methane. CoB–CoM heterodisulfide reductase is a unique disulfide reductase with an essential role in energy metabolism that catalyzes the reversible reduction of the mixed disulfide (CoM-S-S-CoB) of two methanogenic thiol coenzymes, designated coenzyme M and coenzyme B^38, 39^. These enzymes were more abundant in CWS1 than in CWS2 and CK (Fig. 4b). The abundant common enzyme expression in CWS1 indicates that the addition of higher straw biomass could be advantageous for common methanogenic pathways. However, the corresponding methane generation pathways are depicted in Fig. 5. Based on the composition of the methanogenic communities in the present study, *Methanosarcinaceae* was the dominant family and was significantly more abundant in the co-digesters than in the CK. As a result, straw biomass addition to coal can influence the important enzymes from *Methanosarcinaceae* and *Methanobacteriaceae*, which are primarily involved in both the aceticlastic and hydrogenotrophic pathways^40^.

The KEGG module recognized by M-numbers is a group of distinct functional units representing the main metabolic pathways. Figure 4c illustrates the four modules associated with the methanogenic processes. Module M00567 represents the conversion of CO_2_ to methane and was highly abundant in all three reactors, namely, CWS1, CWS2, and CK. The second most abundant module, M00357, was responsible for acetate conversion to CH_4_, followed by M00563 (methyl compound to methane) and finally M00356 (conversion of methanol to methane). However, when comparing the different reactors, all these modules were significantly more abundant in CWS1 than in CWS2 and CK (Fig. 4C). However, considering the methanogenic community, *Methanosarcinaceae* was the main methanogen responsible for these methanogenic processes. The acetoclastic pathway was the primary pathway proposed by^41^ for *Methanosarcinaceae* to generate methane in a syntrophic way instead of via the hydrogenotrophic pathway.

### Succession of metabolic intermediates by LC‒MS

Liquid samples were extracted from each digester during the methanation process to analyze the nontargeted metabolites. Previously, diverse macromolecular compounds, such as aromatics, alkanes, phenols and aliphatics, were observed within the liquid phase of coal biodegradation^15, 52, 53^. In this investigation, a novel exploration was carried out, examining specific metabolic intermediates emerging from the co-degradation of straw biomass and coal. Heatmap analysis, partial least squares discriminant analysis and a Venn diagram showed significantly different results in this study between co-digestion and mono-digestion, as shown in Fig. S3. Table 3 shows the relative abundance of the liquid phase products of co-digestion and coal mono-digestion. The relative abundance of aromatic compounds such as 4-(2-chloroanilino)-4-oxobutanoic acid in CWS1, CWS2, and CK appeared to be 12.10%, 16.49%, and 0.01%, respectively. Phthalic acid was the second most prevalent aromatic compound in CWS2 (12.77%), CWS1 (10.23%), and CK (0.03%). Furthermore, different aromatic and phenolic compounds, such as 4-hydroxybenzoic acid, hexahydro-6,7-dihydroxy-5-(hydroxymethyl)-3-(2-hydroxyphenyl)-2 H-pyrano[2,3-d]oxazol-2-one, indoleacrylic acid, and cinnamic acid, were detected at different percentages across the treated reactors. Remarkably, among these compounds, cinnamic acid was highly abundant in CK (12.72%) compared to the other reactors. During coal biodegradation, aromatic and phenolic compounds are generated by hydroxylation and acylation processes, indicating that the bioavailability of coal increases microbial activity.

**Table 3:** Relative abundance of the top thirty metabolic intermediates identified by LC‒MS during methane generation from co-digestion and mono-digestion processes.

S.no	Metabolite	Retention time	M/Z	Formula	CWS1	CWS2	CK
1	5-Aminopentanoic acid	0.79	118.09	C_5_H_11_NO_2_	0.48	0.54	3.58
2	(R)-(+)-2-Pyrrolidone-5-carboxylic acid	1.37	130.05	C_5_H_7_NO_3_	2.33	2.54	4.53
3	Pantothenic Acid	2.28	220.12	C_9_H_17_NO_5_	0.76	0.59	0.77
4	Succinic Acid	1.69	117.02	C_4_H_6_O_4_	1.16	1.27	0.98
5	FAPy-adenine	1.15	136.06	C_5_H_7_N_5_O	1.79	2.29	1.18
6	Adenosine	0.92	268.10	C_10_H_13_N_5_O_4_	0.73	0.80	0.88
7	Betaine	0.65	118.09	C_5_H_11_NO_2_	0.37	1.58	6.33
8	L-2-Amino-4-methylenepentanedioic acid	0.87	124.04	C_6_H_9_NO_4_	1.10	1.48	0.37
9	6-Hydroxyhexanoic Acid	4.61	131.07	C_6_H_12_O_3_	0.56	0.29	1.72
10	(3b,20R,22R)-3,20,27-Trihydroxy-1-oxowitha-5,24-dienolide 3-glucoside	3.77	635.34	C_34_H_50_O_11_	0.00	0.00	1.41
11	L-Valine	0.86	118.09	C_5_H_11_NO_2_	1.47	1.26	7.29
12	Lys Leu	1.76	260.20	C_12_H_25_N_3_O_3_	0.80	0.65	0.53
13	Indoleacrylic acid	2.62	188.07	C_11_H_9_NO_2_	0.46	0.79	4.20
14	Cinnamic acid	2.14	166.09	C_9_H_8_O_2_	1.51	2.40	12.72
15	Hypoxanthine	1.31	137.05	C_5_H_4_N_4_O	0.68	0.83	1.64
16	Piperidine	1.76	86.10	C_5_H_11_N	2.24	2.04	5.77
17	Norleucine	1.84	132.10	C_6_H_13_NO_2_	11.91	10.26	40.94
18	2-Hydroxycinnamic acid	1.76	182.08	C_9_H_8_O_3_	1.42	1.65	2.69
19	P-Coumaric acid	0.90	182.08	C_9_H_8_O_3_	0.65	0.82	1.14
20	Ile Ile	3.02	245.19	C_12_H_24_N_2_O_3_	1.87	1.33	0.46
21	Daunosamine	0.86	130.09	C_6_H_13_NO_3_	2.57	1.67	0.53
22	Suberic acid	4.91	173.08	C_8_H_14_O_4_	11.80	6.77	0.08
23	Dodecanedioic Acid	5.83	229.14	C_12_H_22_O_4_	1.17	0.82	0.02
24	Adipic Acid	3.23	145.05	C_6_H_10_O_4_	4.35	3.17	0.03
25	4-Hydroxybenzoic Acid	5.64	137.02	C_7_H_6_O_3_	7.59	4.57	0.03
26	Hexahydro-6,7-dihydroxy-5-(hydroxymethyl)-3-(2-hydroxyphenyl)-2 H-pyrano[2,3-d]oxazol-2-one	4.08	280.08	C_13_H_15_NO_7_	5.51	8.05	0.02
27	2-dehydropantoate	3.69	145.05	C_6_H_10_O_4_	3.37	3.75	0.03
28	Deoxyadenosine	1.93	252.11	C_10_H_13_N_5_O_3_	9.01	8.52	0.06
29	4-(2-chloroanilino)-4-oxobutanoic acid	5.14	228.04	C_10_H_10_C_l_NO_3_	12.10	16.49	0.01
30	Phthalic Acid	3.87	165.02	C_8_H_6_O_4_	10.23	12.77	0.03

Furthermore, the hydroxylation of complex macromolecules is assumed to play an important role in the degradation of coal, and further reactions give rise to subsequent metabolites such as fatty acids^19^. Notably, aromatic compounds with 30 carbon atoms undergo transformation in the presence of terminal electron acceptors such as iron, nitrite, and sulfate^19^. The syntrophic association of hydrogenotrophic methanogens and sulfate-reducing bacteria could facilitate the degradation of these compounds^54, 55^. Our results are consistent with those of a study conducted by Strąpoć and colleagues showing that aromatic hydrocarbons are generated during coal biodegradation and are further utilized by methanogens to produce methane^2^.

Aliphatic compounds, on the other hand, were generated in all reactors at different abundances. Notably, norleucine (a nonproteinogenic amino acid) exhibited a high abundance in CK (40.94%), followed by CWS1 (11.91%) and CWS2 (10.26%). High concentrations of L-valine (7.29%) and Betaine (6.33%) were also found in the CK reactor compared to the other reactors. The specific role of norleucine in the context of coal bioconversion remains unclear and has not been documented explicitly. However, its prevalence could be related to specific microorganisms or enzymatic processes linked to coal biodegradation. Betaine is an alkaloid that contributes to maintaining the alkalinity of the medium and has also been found during the degradation of Dananhu low-rank coal^56^. Other aliphatic compounds, such as suberic acid, adipic acid, and deoxyadenosine, were more abundant in co-digesters than in CK.

However, the high abundance of such aromatic and phenolic compounds in CWS2 and CWS1 indicates high degradation of coal and wheat straw, which can be further transformed by fermenting bacteria into intermediate products such as acetic acid, organic acids, long-chain fatty acids and other simple organic compounds^57^. During coal methanogenesis, the multifarious composition and irregular structure of coal interacted with microbial communities, and their diverse metabolic activities make the bioconversion process of coal very complex. Moreover, the understanding of the impacts of coal intermediates and the mechanism of coal bioconversion during methanogenesis is still limited^43^. The organic intermediate of coal includes a mixture of different hydrocarbons and heterocyclic structures^58^. Further degradation of these compounds leads to the production of H_2_, CO_2_, acetate, and methanol as methanogenic substrates^59^. In correlation with the identified microbial communities, this study revealed unique metabolic characteristics not previously documented during methane generation from coal and straw biomass. The metabolic analysis also suggested that during the biodegradation process, the microbial behavior differed between single-coal digestion and co-digestion with straw biomass.

**Figure 6:**
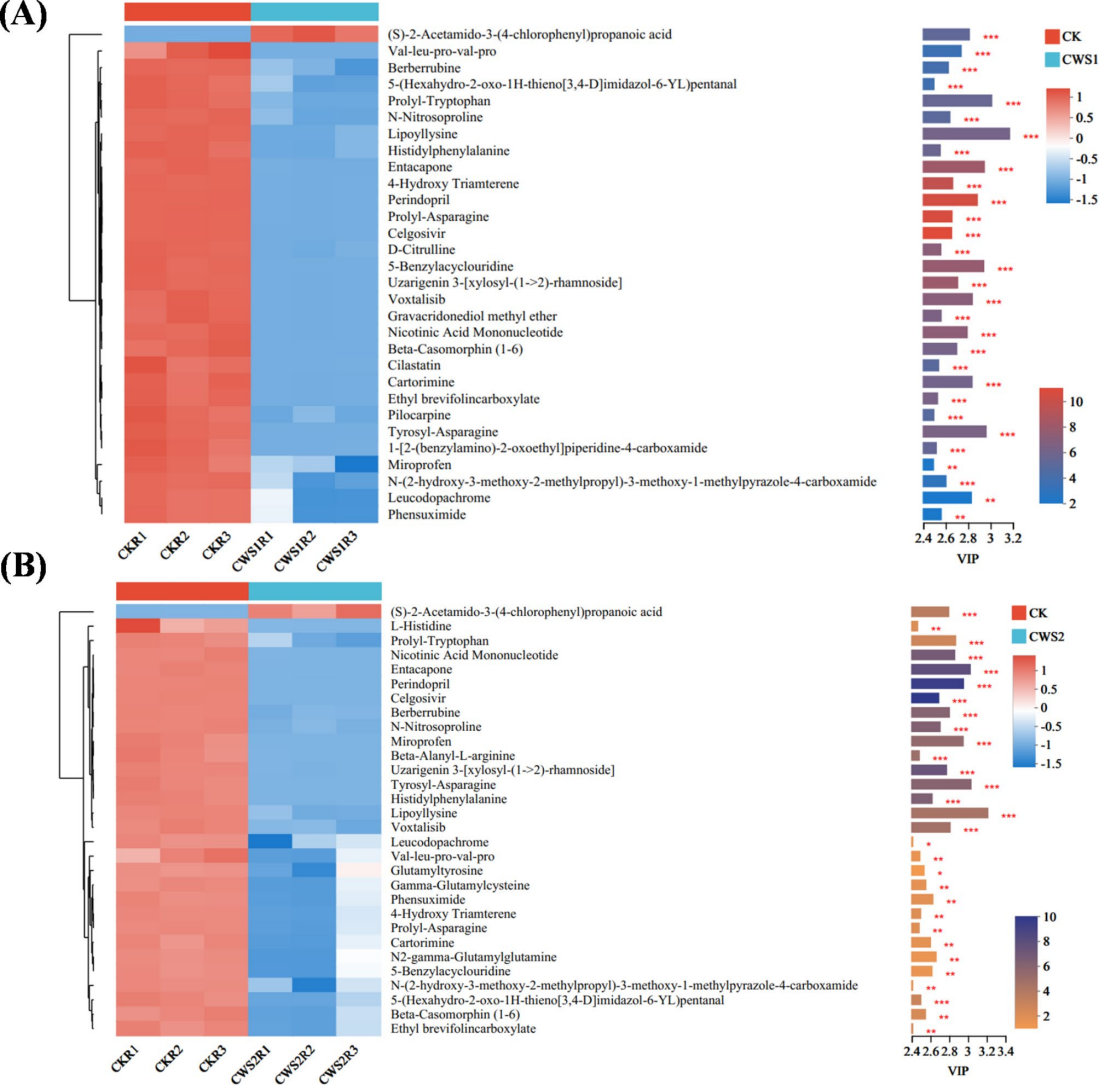
Expression of VIP metabolites and their significance in the two groups. The VIP analysis was performed in the online platform of Majorbio Cloud Platform (www.majorbio.com) using ropls(R); scipy(Phython) Version 1.6.2; Version 1.0.0 (https://www.bioconductor.org/packages/release/bioc/html/ropls.html) (**A**) shows the combination of CK and CWS1, and (**B**) shows the combination of CK and CWS2.

Metabolites demonstrating a VIP value exceeding 1.0 in the OPLS-DA model, coupled with a statistically significant threshold of *P* < 0.05 determined by a t test, were identified as differentially expressed metabolites across assays^60, 61^. Figure 6A and B delineate the top 30 discriminating metabolites, each meeting the stringent criteria of VIP > 1 and *p* < 0.05, which were consistently observed across all the treatment groups. These selected metabolites are depicted via a heatmap reflecting their abundance and corresponding statistical significance. The integration of both cluster heatmap visualization and VIP bar chart representation allowed for a comprehensive illustration of metabolite expression dynamics within the individual treatment groups, concurrently elucidating the statistical significance denoted by p values and VIP scores derived from multivariate statistical analysis. This graphical depiction effectively elucidated the profound significance of the identified differentially expressed metabolites and revealed their distinct expression patterns within the studied context.

## Conclusion

This investigation explores the potential of methane generation and metabolic processes involved in the co-digestion of coal and wheat straw. Wheat straw used as co-substrate with coal had significant impacts on methane generation. *Methanosarcinaceae* and *Methanobacteriaceae* were the primary methanogens, evenly distributed throughout the co-digestion process. The predicted relative abundance of enzymes associated with common and hydrogenotrophic metabolic pathways was higher than that of enzymes linked to aceticlastic and methylotrophic metabolic pathways. Additionally, liquid phase analysis showed that the metabolic intermediates from wheat straw and coal co-degradation influenced the methane generation.

## Supplementary Information

Supplementary Material 1.

## Data Availability

The datasets generated and analyzed during the current study are available in the NCBI short read archive (SRA) under the Bioproject accession number PRJNA1086209, with biosample accessions SRX23920174, SRX23920173, and SRX23920172.
