# Supplementary material for: Coal-straw co-digestion-induced biogenic methane production: perspectives on microbial communities and associated metabolic pathways
Source: Sci Rep. 2024 Nov 4;14:26554. doi: 10.1038/s41598-024-75655-z (PMC11532504; doi:10.1038/s41598-024-75655-z)
Supplement: Supplementary file 1 — Supplementary Material 1. [file 41598_2024_75655_MOESM1_ESM.docx]

**Supplementary Materials**

**Coal-Straw Co-digestion-induced Biogenic Methane Production: Perspectives on Microbial communities and Associated Metabolic Pathways**

Sohail Khan^1,3^, Ze Deng^2*^, Bobo Wang^1^, Zhisheng Yu^1,3*^

**^1^**College of Resources and Environment, University of Chinese Academy of Sciences, 19 A Yuquan Road, Beijing 100049, P.R. China

^2^PetroChina Research Institute of Petroleum Exploration and Development, Beijing 100083, P.R. China

^3^RCEES-IMCAS-UCAS Joint-Laboratory of Microbial Technology for Environmental Science, Beijing 100085, P.R. China

*Corresponding Authors: Zhisheng Yu and Ze Deng

Prof. Dr. Zhisheng Yu

College of Resources and Environment, University of Chinese Academy of Science, 19 A Yuquan Road, Beijing 100049, P.R. China

Email: [yuzs@ucas.ac.cn](mailto:yuzs@ucas.ac.cn)

Fig S1. NMDS analysis of microbial communities based of OTUs (a) bacterial community; and (b) archaeal community.


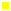

Fig. S2. Heatmap analysis of genes encoding essential bacterial enzymes (A), Genes encoding essential archaeal enzymes (B). The analysis was done using PICRUSt2 version 2.2.0; OUT statistics, KEGG orthologys (KO), EC, COG and MetaCyc metabolic pathway prediction (https://github.com/picrust/picrust2/)

Fig. S3. Heatmap analysis (A), Partial Least Squares Discriminant Analysis (PLS-DA)(B), and Venn diagram highlighting the distribution and difference in metabolic components generated in co-digestions reactors CWS1 and CWS2, and CKR (Coal alone).
